# AMPK: An Epigenetic Landscape Modulator

**DOI:** 10.3390/ijms19103238

**Published:** 2018-10-19

**Authors:** Brendan Gongol, Indah Sari, Tiffany Bryant, Geraldine Rosete, Traci Marin

**Affiliations:** 1 Department of Medicine, University of California, San Diego, CA 92093, USA; brengong@gmail.com; 2 Department of Cardiopulmonary Sciences, School of Allied Health Professions, Loma Linda University, Loma Linda, CA 92350, USA; isari@llu.edu (I.S.); tprescott@llu.edu (T.B.); grosete@llu.edu (G.R.); 3 Department of Health Sciences, Victor Valley College, Victorville, CA 92395, USA

**Keywords:** AMPK, epigenetics, chromatin remodeling, histone modification, DNA methylation

## Abstract

Activated by AMP-dependent and -independent mechanisms, AMP-activated protein kinase (AMPK) plays a central role in the regulation of cellular bioenergetics and cellular survival. AMPK regulates a diverse set of signaling networks that converge to epigenetically mediate transcriptional events. Reversible histone and DNA modifications, such as acetylation and methylation, result in structural chromatin alterations that influence transcriptional machinery access to genomic regulatory elements. The orchestration of these epigenetic events differentiates physiological from pathophysiological phenotypes. AMPK phosphorylation of histones, DNA methyltransferases and histone post-translational modifiers establish AMPK as a key player in epigenetic regulation. This review focuses on the role of AMPK as a mediator of cellular survival through its regulation of chromatin remodeling and the implications this has for health and disease.

## 1. Introduction

Epigenetic regulation gives rise to a spectrum of cellular phenotypes observed in a single organism independent of primary DNA sequence. Such regulation is hereditable and stable, as occurs in the determination of cell type, but also transient, producing a particular phenotypic outcome to ensure survival [1,2,3]. Influencing gene expression, epigenetics promotes organismal adaption by offering substantial functional variability in response to environmental stimuli [1,2,3]. This regulation occurs, in part, through nucleosomal remodeling as a result of histone, DNA, and DNA-binding protein modifications that include: phosphorylation, acetylation, *O*-GlcNAcylation, ribosylation, and methylation. Such signature modifications or marks characterize nucleosome remodeling and determine the degree of gene activation or silencing. At a fundamental level, stressors such as nutrient deprivation or heightened physical activity trigger dynamic epigenetic markings that orchestrate adaptive gene regulation to improve survivability [4,5,6,7].

AMP-activated protein kinase (AMPK), a master regulator of energy homeostasis and a key mediator of adaptation and cell survival, is activated by conditions that produce energy deprivation such as hypoxia, exercise, nutrient starvation, and infection [8]. AMPK functions as a heterotrimeric serine/threonine protein kinase composed of a catalytic α-subunit, a scaffolding β-subunit, and a regulatory γ-subunit. Both α and β-subunits exist in two isoforms (α1, α2, and β1, β2), while the regulatory γ-subunit exists in three isoforms (γ1, γ2, γ3). The combination of α, β, and γ isoforms can form 12 different heterotrimeric complexes, each demonstrating unique functions and subcellular or tissue-specific distributions [9,10,11]. Once activated, AMPK phosphorylates targets with well-defined consensus sequences to regulate bioenergetics by modulating metabolic pathways that promote ATP production and limit energy expenditure [12]. Such roles of AMPK in cellular homeostasis are facilitated by gene regulation as a result of chromatin conformational dynamics. To that end, AMPK orchestrates these changes by phosphorylating several histones and proteins involved in nucleosome remodeling that enhance mitochondrial biogenesis and function [13]. Additionally, during acute metabolic stressors, such as fasting or exercise, AMPK associates with chromatin at promoters of genes involved in lipid and glucose metabolism [14].

Chronic AMPK activation plays a role in cellular and organismal inheritance, evidenced by its multi-isoform 2R-ohnologue characteristics, which are often evolutionarily conserved in gene coding regions to support basic survival functions and to increase the possibility for complex tissue diversity and adaptation [15,16,17]. Such adaptation for survival early in life initiates epigenetic programming that correlates with AMPK activation and determines predisposition to disease. For example, insults during development, such as placental insufficiency or maternal metabolic disorders, increase the probability of adverse metabolic disorders in later stages of life, such as diabetes mellitus, metabolic syndrome, insulin resistance, hypertension, vascular disease, and cancer [18]. Furthermore, metabolic disorders, such as maternal obesity, are often associated with reduced AMPK expression or activity and concomitant loss of its epigenetic mechanisms associated with adaptation and survival [19,20]. Although the mechanistic basis for this regulation, as well as its implications for inheritance, is still under exploration, these collective observations point to a fundamentally important role of AMPK as an epigenetic regulator.

## 2. Histone Modification

Histones are nuclear-localized, primarily positively charged proteins. However, a number of histone splice variants have been described with broad cellular functions and localizations [21,22]. Within the nucleus, histones are packaged into an octamer consisting of two H2A, H2B, H3, and H4 [23]. These octameric histone complexes associate via charge–charge interactions with DNA and make up a nucleosome, which is linked to other nucleosomes by histone H1. Given their tight association with DNA, histones serve as mediators between stress response signaling cascades and the regulation of nucleosomal structure that ultimately influence gene expression and cellular survival [24]. AMPK, both directly and indirectly, regulates the post-translational modification (PTM) status of histones that play a major role in the regulation of nucleosome structure.

### 2.1. Histone Phosphorylation

**Figure 1:**
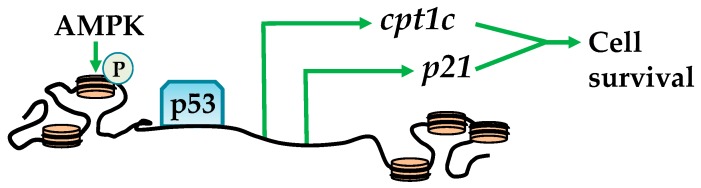
AMPK (AMP-activated protein kinase) promotes cell survival through histone phosphorylation. AMPK phosphorylates H2B to promote chromatin relaxation at tumor protein p53 (p53) recognized promoters and transcription of *carnitine palmitoyltransferase 1C* (*cpt1c*) and *cyclin-dependent kinase inhibitor* (*p21*) to enhance cell survival.

Bioinformatic analysis has identified several histones that contain an AMPK phosphorylation consensus sequence. These include: H1FX, H2AFX, H2AFY, H2AFY2, H2AFZ, H2BFM, and H3F3B [25], suggesting that AMPK plays an important role in epigenetic regulation through histone phosphorylation. Ultimately, such histone phosphorylation promotes cross-talk between epigenetic regulators to facilitate spatio-temporal nucleosome structural changes that influence transcriptional machinery [24]. For example, activation by glucose deprivation or UV radiation results in the colocalization of AMPK and phosphorylating serine 36 in H2B (H2B^S36^) throughout the *carnitine palmitoyltransferase 1C* (*CPT1C*) and *cyclin-dependent kinase inhibitor* (*p21*) promoter regions and gene bodies. This exposes TP53 or tumor protein (p53) DNA binding sites and subsequent association with RNA polymerase II (Figure 1) [26,27]. These events enhance CPT1C and p21 expression, which play a role in cellular survival via activating autophagy and transporting long-chain fatty acids to the mitochondria, collectively enhancing β-oxidation and energy production [28,29,30]. Related to cellular survival, charged multivesicular body protein 1B (CHMP1b) is phosphorylated by AMPK, forms a “shell” around nucleosomes enriched with H3 phosphorylation and acetylation, and influences gene transcription [25]. This might play a role in the transition of active and inactive nucleosome regions and potentially heritable epigenetic marks [31].

### 2.2. Histone Acetylation

Lysine acetylation of the N-terminal tails of histones disrupts the charge–charge interactions between DNA and histone tails, producing a euchromatin, a relaxed and active, chromatin state. This reaction changes histone–DNA associations, histone–histone associations between adjacent nucleosomes, and histone–regulatory protein interactions [32]. Histone acetylation occurs via transfer of an acetyl group from acetyl coenzyme-A (acetyl-CoA) to the to the ε-ammonium (NH_3_^+^) group of lysine catalyzed by histone acetyltransferases (HATs). HAT families are diverse and promote a spectrum of interactions and functions. Histone deacetylation restores the DNA-histone interaction promoting a heterochromatic, a condensed and silent chromatin state [33]. Antagonistically, histone deacetylation, catalyzed by histone deacetylases (HDACs), restores the DNA-histone interaction promoting a euchromatic chromatin state [33]. AMPK regulates the activity of both HATs and HDACs by influencing cofactor or substrate availability through direct phosphorylation.

**Figure 2:**
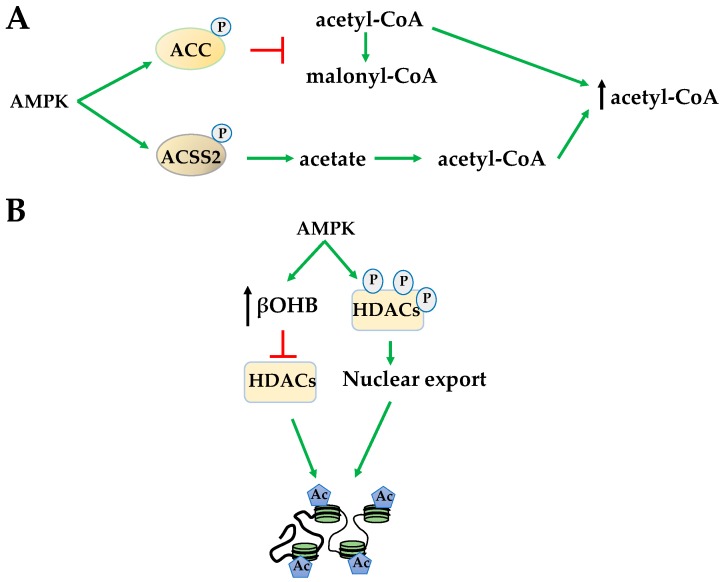
AMPK activation increases acetyl-CoA and promotes histone acetylation. (**A**) Through phosphorylation, AMPK inhibits acetyl-CoA carboxylase (ACC) while activating acetyl-CoA synthetase short-chain family member 2 (ACSS2) to increase acetyl-CoA availability for acetylation; (**B**) AMPK increases β-hydroxybutyrate (βOHB) to inhibit histone deacetylases (HDACs) and promotes HDAC nuclear export via hyperphosphorylation. Both of these events increase histone acetylation.

Indirectly, 5-Aminoimidazole-4-carboxamide ribonucleotide (AICAR) activation of AMPK affects global HAT activity by increasing available acetyl-CoA levels through several mechanisms. AMPK phosphorylates acetyl-CoA carboxylase (ACC) to prevent the conversion of acetyl-CoA to malonyl-CoA increasing available acetyl donating groups for HATs (Figure 2A). AMPK also increases the formation of acetyl-CoA by phosphorylating acetyl-CoA synthetase short-chain family member 2 (ACSS2), causing its nuclear translocation for the conversion of acetate, the byproduct of HDAC histone deacetylation, to acetyl-CoA (Figure 2A). This mechanism has been shown to increase HAT acetylation of H3 at transcription factor EB (TFEB)-responsive promoters to activate genes important for autophagy and lysosomal function [34,35]. Yet, in general, increased acetyl-CoA levels in response to AICAR results in increased H3 acetylation at lysine 14 (H3^K14^) and H4 acetylation at lysines 5,8,12,16 (H4^K5,8,12,16^) [36]. AMPK activated by metformin regulates histone acetylation in a metabolic state-dependent manner. For example, metformin reduces H3 acetylation by decreasing bioavailability of mitochondrial acetyl-CoA in breast cancer, bringing acetylation status to normal in promoters of cancer-specific genes [37].

AMPK also regulates substrate availability of HDACs. The class III HDACs, sirtuins (SIRTs), couple deacetylation with nicotinamide adenine dinucleotide (NAD^+^) hydrolysis to produce *O*-acetyl-ADP-ribose [38]. AMPK indirectly activates SIRT1, in part, by increasing the NAD^+^/NADH ratio [38,39]. Once activated, SIRT1 plays a fundamental role in chromatin organization by interacting with and deacetylating a variety of transcription factors and coregulators [40]. However, in contrary to activating the SIRTs, AMPK indirectly inhibits class I and II HDACs by increasing β-hydroxybutyrate (βOHB) during fatty acid oxidation. βOHB acts similarly to the HDAC inhibitor butyrate, increasing global histone acetylation (Figure 2B) [41]. However, in addition to globally inhibiting class I and class II HDACs, AMPK activation also promotes HDACs 4, 5, and 7 hyperphosphorylation and translocation from the nucleus (Figure 2B) [42]. Both of these events increase global histone acetylation. However, despite these global effects, the regulation of gene expression is often promoter- and gene cluster-specific, suggesting the importance of direct phosphorylation events of AMPK on specific HATs and HDACs.

**Figure 3:**
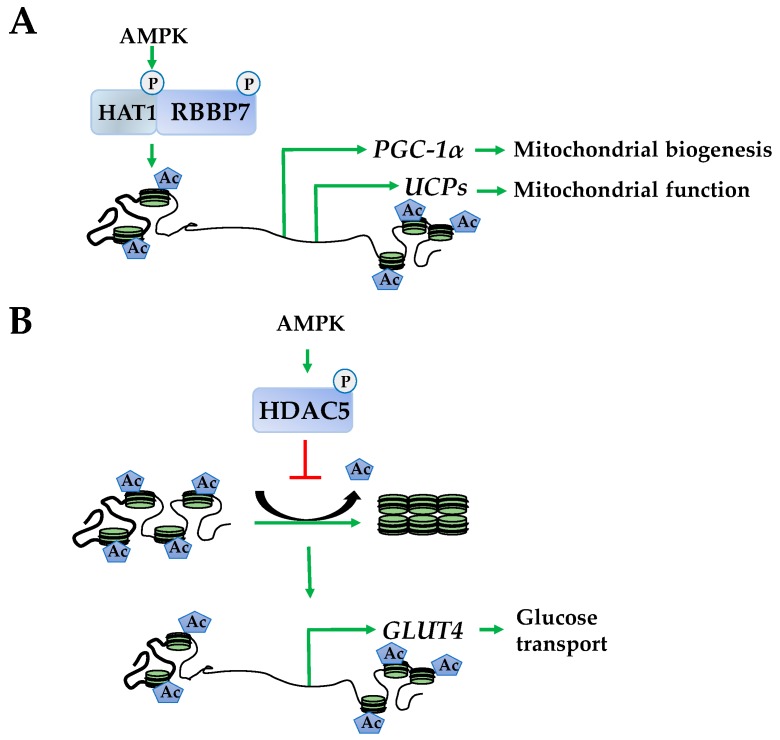
AMPK inhibits histone deacetylase activity to promote histone acetylation. (**A**) AMPK phosphorylates histone acetylase1 and retinoblastoma binding protein 7 (RBBP7) to form a complex that acetylates histones at promoters of *peroxisome proliferator-activated receptor gamma coactivator-1α* (*PGC-1α*), *uncoupling proteins* (*UCPs*) to increase mitochondrial biogenesis and function; (**B**) AMPK phosphorylates and inhibits HDAC5 enhancing acetylation at the *glucose transporter type 4* (*GLUT-4*) promoter increasing its transcription.

Investigations into epigenetically regulated networks identified HAT1 and retinoblastoma binding protein 7 (RBBP7) as direct targets of AMPK that dimerize following phosphorylation. This results in enhanced euchromatin structure at the promoters of *peroxisome proliferator–activated receptor gamma coactivator–1α* (*PGC-1α*), *transcription factor A* (*Tfam*), *nuclear respiratory factors 1* and *2* (*NRF1* and *NRF2*), and *uncoupling proteins 2* and *3* (*UCP2* and *UCP3*). The corresponding induction of these genes produces enhanced mitochondrial function (Figure 3A) [13]. In addition to HAT1 and RBBP7, AMPK phosphorylates HDAC5, promoting its dissociation from the promoters releasing its suppressive effects to increase expression of an array of metabolic genes, including glucose transporter type 4 (GLUT-4) (Figure 3B) [43].

Although less characterized, it is likely that AMPK phosphorylates an array of HATs including transcription regulator family member A (SIN3), CREB binding protein (CREBBP), elongator acetyltransferase complex subunits (ELP) 2, 3, and 4, and K(lysine) acetyltransferases (KAT) 2A, 2B, 6A, 6B, 7, and 8, and HDACs 1–5, 8–9, and 10, 11, as well as SIRTs 2, 3, 4, 5, 6, and 7; all of which contain AMPK consensus sequences and are opportune for future study [13].

### 2.3. Histone Methylation

**Figure 4:**
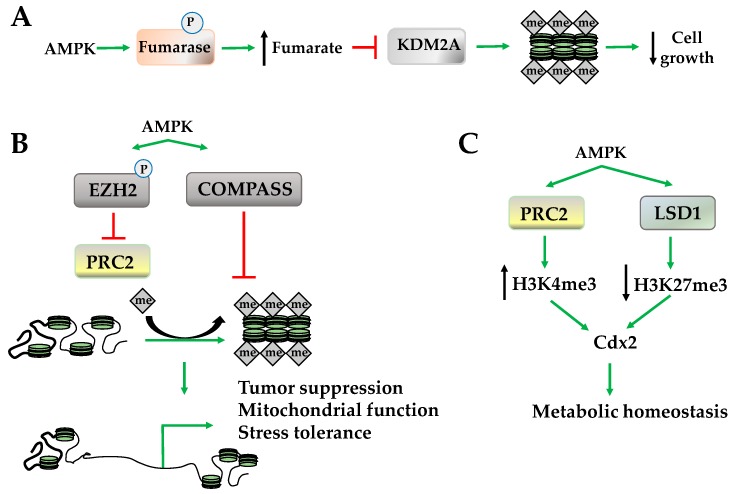
AMPK regulates histone methylation. (**A**) AMPK phosphorylates fumarase increase fumarate, which inhibits lysine-specific demethylase 2A (KDM2A), increasing histone methylation at cell growth; (**B**) AMPK decreases histone methylation through inhibition of polycomb repressive complex 2 (PRC2) and complex proteins associated with set1 (COMPASS complex); (**C**) AMPK modulates histone 3 methylations status to increase caudal type homeobox2 [cell differentiation]) (Cdx2) expression and metabolic homeostasis. LSD1: lysine-specific histone demethylase-1; EZH2: zeste homolog 2.

Histone methylation occurs on lysine and arginine, and its effects on gene expression are dependent upon the site and degree of methylation. For example, methylation of H3^K4^, H3^K36^, and H3^K79^ promotes an active euchromatin state; while methylation of H3^K9^ and H3^K27^ promotes a silent heterochromatic state [44,45,46]. Both lysine-specific and arginine-specific histone methyltransferases use S-adenosyl methionine (SAM) as cofactors and methyl donor catalyzing the transfer of one, two, or three methyl groups. The demethylases, however, serve a variety of functions and include two primary classes: flavin adenine dinucleotide (FAD)-dependent amine oxidase and Fe(II) and α-ketoglutarate-dependent hydroxylase. The regulation of demethylases via α-ketoglutarate, a tricarboxylic acid (TCA) cycle intermediate, suggests that AMPK may play a role in regulating histone methylation and demethylation because AMPK is activated by conditions where TCA cycle intermediates are depleted and, once activated, these intermediates are restored. For example, enhanced amino acid, fatty acid, or glycogen catabolism by AMPK increases α-ketoglutarate, which, in turn, may increase histone demethylase activity [47]. In addition to restoring α-ketoglutarate levels, AMPK phosphorylates and inhibits fumarase a TCA cycle enzyme that converts fumarate to malate. Subsequently, elevated fumarate levels inhibit lysine-specific demethylase 2A (KDM2A), restoring H3^K36^ dimethylation (H3^K36me2^) at promoters of genes mediating cell growth (Figure 4A) [48]. In addition to these indirect effects, AMPK activates the demethyltransferase lysine demethylase 5 (KDM5) and lysine-specific histone demethylase-1 (LSD1) that remove H3^K4^ trimethylation (H3^K4me3^) [49]. Although AMPK activates histone demethyltransferase activity, it inhibits several complexes that regulate methyltransferase activity including the methylation complex polycomb repressive complex 2 (PRC2) and the histone methyltransferase-containing COMPASS complex (complex proteins associated with Set1) [20,50]. While the inhibition of PRC2 occurs through the phosphorylation of histone methyl transferase enhancer of zeste homolog 2 (EZH2), which decreases H2^K27^ monomethylation (H2^K27me1^), inhibition of the COMPASS complex by AMPK decreases H3^K4me3^, a marker of transcriptional activation (Figure 4B) [50,51,52]. Globally, inhibition of PRC2 results in the upregulation of tumor suppressor PRC2 target genes and suppression of tumor growth, while COMPASS complex inhibition is a protective mechanism that halts chromatin marking in the presence of nutrient deficiency to promote stress tolerance (Figure 4B) [20,50,51,52].

However, the orchestration between the methylation and demethylation activities of AMPK is promoter-specific. For example, AMPK recruits PRC2 and LSD1 to the promoter of *caudal type homeobox2* (*Cdx2)*, increasing H3^K4me3^ but not H3^K27me3^ [53]. This increases CDX2 expression, which transactivates solute carrier family 5 member 8 (SLC5A8) to increase immune function and cell survival under several stress conditions (Figure 4C) [54,55]. 

### 2.4. Histone O-GlcNAcylation

**Figure 5:**
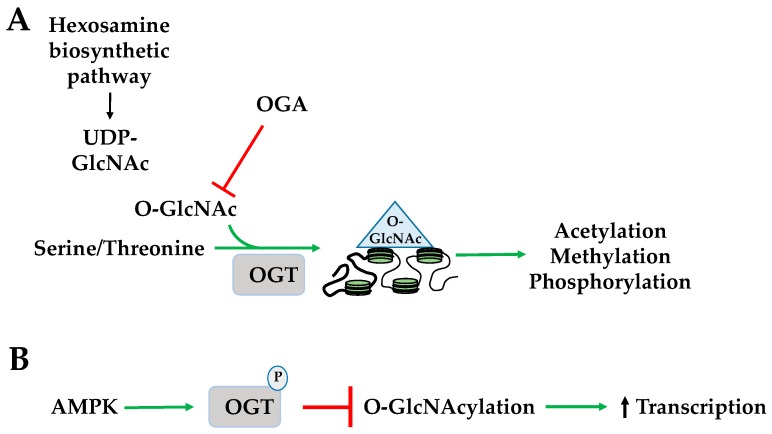
AMPK regulates histone *O*-GlcNAcylation and ADP-ribosylation. (**A**) *O*-GlcNAc transferase adds *O*-GlcNAc to a serine or threonine to produce *O*-GlcNAcylation. Nutrient and stress sensor stimulates hexosamine biosynthetic pathway (HBP) to create UDP-GlcNAc, which is a donor substrate for *O*-GlcNAcylation. Histone *O*-GlcNAcylation regulates acetylation, methylation, and phosphorylation; (**B**) AMPK phosphorylates *O*-GlcNAc transferase and inhibits *O*-GlcNAcylation to increase transcription. OGA: *O*-GlcNAcase; OGT: *O*-GlcNAc transferase.

*O*-GlcNAcylation is the addition of an *O*-linked *N*-acetylglucosamine (*O*-GlcNAc) group to a serine or threonine by *O*-GlcNAc transferase (OGT). The donor substrate for *O*-GlcNAcylation, uridine diphosphate *N*-acetylglucosamine (UDP-GlcNAc), is a product of the hexamine biosynthetic pathway (HBP) in which glucose, fatty acid, amino acid, and ATP metabolism converge implicating its role as a nutrient and stress sensor [56]. *O*-GlcNAcase (OGA) removes *O*-GlcNAc through hydrolysis. The dynamics of histone *O*-GlcNAcylation regulate a variety of activities including other histone PTMs such as acetylation, methylation, and phosphorylation, and is highly sensitive to a spectrum of cellular stressors such as hypoxia, heat shock, and starvation (Figure 5A) [57]. AMPK phosphorylates *O*-GlcNAc transferase (OGT) promoting its dissociation from chromatin. This inhibits its *O*-GlcNAcylation of H2B^S112^ in response to extracellular glucose through the hexosamine biosynthesis pathway (HBP) and promotes H2B^K120^ mono-ubiquitination and transcriptional activation (Figure 5B) [56].

### 2.5. Histone Ribosylation

Histone ADP-ribosylation can occur as addition of mono- or poly-ADP-ribosylation units by mono-ADP ribosyltransferases (ARTs) or poly-(ADP-ribose) polymerases (PARPs), respectively. NAD^+^ serves as the main source for histone ADP-ribosylation, which can occur on many amino acids restructuring chromatin into a euchromatin state [58,59]. AMPK phosphorylates and activates PARP1 [60,61]. Substrates of PARP1 include PARP1 itself and the tail of histones H1, H2A, H2B, H3, and H4, creating a poly(ADP-ribose)ylated mark at transcription start sites in transcriptionally necessary genes during metaphase [62]. Contrarily, when PARP1 is not active as a polymerase, it binds to DNA and promotes a heterochromatin state [59]. This is underscored by AMPK’s phosphorylation of PARP1, causing its dissociation from the B-cell lymphoma protein 6 (Bcl-6) to increase its expression [61].

## 3. DNA Modification

More abundantly enriched in promoter regions, cytosine-guanine dinucleotides (CpG) are susceptible to methylation at the 5’ cytosine position by a family of DNA methyltransferases (DNMTs). Each DNA methyltransferase member has a slightly different functional role in the global regulation of global DNA methylation. While DNMT 3a and 3b create the methyl “marks” that are carried through mitosis, DNMT1 maintains these methylation marks and regulates the dynamics of DNA modification and nucleosomal remodeling [63]. Following methylation, a number of functional changes occur. Due to its electron donating effects, methylation weakens Watson–Crick base pairing and recruits methyl-CpG binding domain proteins (MBDs), HDACs, and transcriptional repressors that collectively organize chromatin into a heterochromatic, a transcriptionally inactive conformation. These CpG methylations are highly dynamic- and stimulation-dependent. Removal of the CpG methylation requires oxidation of 5-methycytosine (5-mC) to 5-hydroxymethylcytosine (5-hmC), which is then converted to 5-formylcytosine (5-fC), and 5-carboxylcytosine (5-caC) followed by complete removal of the functional group by ten-eleven translocation hydroxylases (TETs) [64,65]. Although TETs are required to actively remove CpG methyl groups, the process occurs passively and semi-conservatively during DNA replication [66].

### DNA Methylation

**Figure 6:**
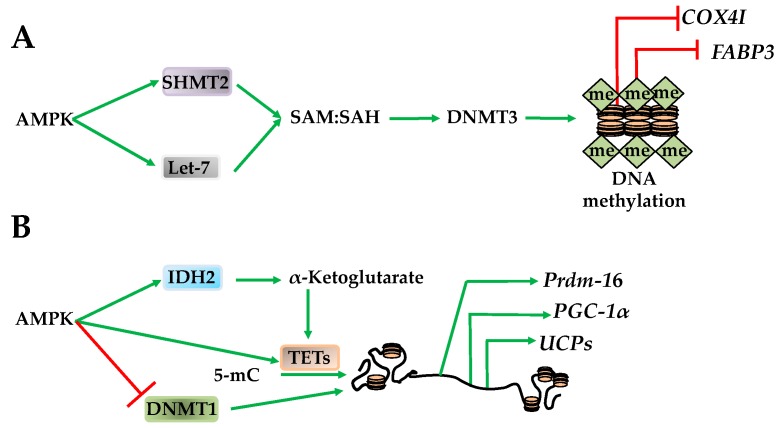
AMPK regulates DNA methylation. (**A**) AMPK activates serine hydroxymethyltransferase 2 (SHMT2) and Let-7 to increase SAM:SAH ratio providing substrate for DNA methyltransferase 3 (DNMT3) methylation of promoters such as *COX4I* and *FABP3*; (**B**) AMPK directly regulates isocitrase dehydrogenase 2 (IDH2) to yield α-ketoglutarates to promote ten-eleven translocation hydroxylases (TETs) activation. AMPK likely also directly regulates TETs catalyzing the conversion of 5-mc to unmethylated cytosine while inhibiting DNA methyltransferase 1 (DNMT1). These actions result in decreased promoter methylation of *PR domain containing 16* (*Prdm16*), *PGC-1α*, and the *uncoupler proteins*.

AMPK regulates global methylation by changing the substrates required for DNMT and TET activity. For example, SAM, the methyl donor for DNMT3a and DNMT3b, is converted to S-adenosylhomocysteine (SAH) upon CpG methylation [67]. Therefore, while SAM is required for DNMT3a and DNMT3b activity, SAH has an opposing inhibitory effect [67]. Supporting DNMT3a and DNMT3b activation, AMPK increases mitochondrial function and serine hydroxymethyltransferase 2 (SHMT2). Once activated, SHMT2 facilitates the one carbon transfer from folate to SAH, making SAM, and increasing the SAM/SAH ratio [67]. AMPK also increases the SAM/SAH ratio by transactivating let-7 micro RNA that subsequently degrades H19, relieving a direct inhibitory effect on S-adenosylhomocysteine hydrolase (SAHH). Subsequent activation of SAHH enables DNMT3b activation [68]. Taken together, these studies indicate that AMPK increases the SAM/SAH ratio that has an activating effect on DNMT3a and DNMT3b. These AMPK-dependent shifts in methylation also correlate to metabolic adaptive situations. For example, promoter methylation of *cytochrome C oxidase subunit 4I1* (*COX4I*) and *fatty acid binding protein 3* (*FABP3*) increases during exercise, while *peroxisome proliferator-activated receptor δ* (*PPARδ*) promoter methylation increases after fasting (Figure 6A) [69]. However, the overall effect AMPK has on DNA methylation is likely to be stimulation-, tissue-, promoter-, and DNMT isoform-specific. Paralleling its role as an epigenetic metabolic regulator in histone acetylation through HAT1 and RBBP7, AMPK also phosphorylates DNMT1^S730^, inhibiting methyl CpG in *PGC-1α*, *Tfam*, *NRF1*, *NRF2*, *UCP2* and *UCP3* promoters [13]. This results in improved mitochondrial function, a requirement for cell survival [13,70].

In addition to the DNMTs, AMPK also regulates TETs, which, in turn, govern loci-specific CpG methylation. The effect of AMPK on TETs occurs through its regulation of TCA cycle intermediates. For example, AMPK regulates isocitrase dehydrogenase 2 (IDH2) to increase the levels of α-ketoglutarate, an activator of TET1–TET4 resulting in CpG demethylation [47,71]. Activation of TET1–TET4 results in transactivation of the PR domain containing 16 (Prdm16) gene in progenitor cells that promotes brown adipogenesis, therefore supporting AMPK’s role as an epigenetic regulator of metabolic function (Figure 6B) [47].

## 4. Approaches to Elucidating the AMPK-Modulated Epigenetic Landscape

Although biochemical studies have resolved a number of mechanistic insights into the role AMPK plays in the epigenetic regulation of chromatin structure, the study of AMPK on epigenetic function is still in its infancy. A number of predictive algorithms and studies have identified a potential role of AMPK on a number of epigenetic regulators. However, the role they may play under different physiological stimulations and their tissue specificity are still unknown. Because of the interconnected nature between regulatory cascades, the study of AMPK on epigenetic function in a disease-relevant capacity requires systems biological and bioinformatics approaches.

**Figure 7:**
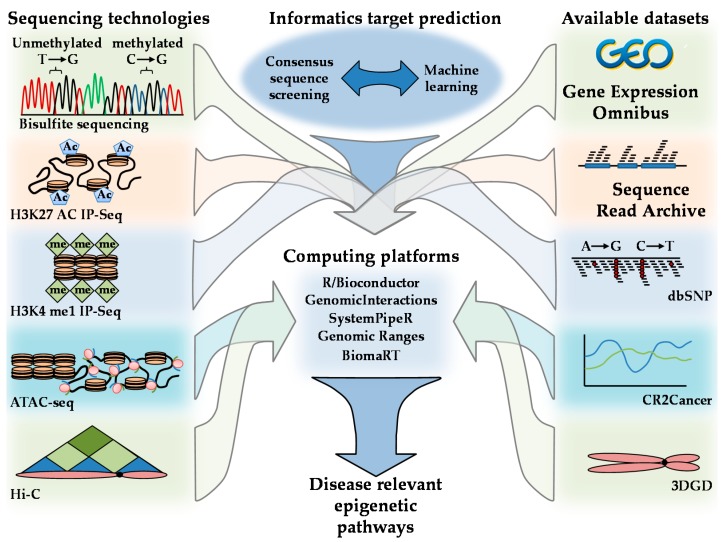
Methods for resolving disease-relevant AMPK-regulated epigenetic networks. Developed sequencing technologies are listed on the left-hand figure panels. Publicly available data repositories are listed on the right. Computing platforms R and relevant Bioconductor packages including Genomic interactions, SystemPipeR, Genomic Ranges, and BioMart can be used to integrate sequencing technologies, publicly available datasets, and AMPK target predictions as illustrated in the center panels.

Scientific advances in the computational and Big Data arenas have resulted in novel experimental approaches, databases, and bioinformatics techniques that can be used in the exploration of AMPK epigenetic-regulated signaling pathways. Examples of tools used to explore chromatin characteristics include H3^K27^ acetylation H3^K4^ and monomethylation (H3^K4me1^) immunoprecipitation sequencing (IP-Seq); assay for transposase-accessible chromatin using sequencing (ATAC-seq); assay for transposase-accessible chromatin using sequencing (ATAC-seq); and Hi-C [72]. The relationship between DNA, histone modifications, histone remodeling, followed by Hi-C structural mapping, provides an integrative understanding of how AMPK can influence chromatin architecture. The identification of AMPK-regulated networks can be explored using consensus sequence mapping and machine learning in computing environments such as R/Bioconductor computing [25,73,74,75]. The expression of effected genes and loci and their application of these loci to disease-relevant stimuli can be further explored via cross-referencing with expression profiles housed in the Gene Expression Omnibus (GEO) database, Sequence Read Archive (SRA), Single Nucleotide Polymorphism database (dbSNP), 3D Genome database C (3DGD), and CR2Cancer [76,77,78,79]. Integration of these datasets can provide a comprehensive picture of the influence AMPK has on epigenetic signaling cascades in addition to their genetic loci and disease-specific regulation (Figure 7).

## 5. Conclusions

AMPK’s role as an epigenetic landscape modulator is underscored by its multifunctional kinase effects that regulate histones and epigenetic enzymes to mediate histone and DNA modifications. AMPK is crucial for cell survival and adaptation as evident by its activation upon nutrient depletion and stressors resulting in histone phosphorylation, acetylation, methylation, *O*-GlcNAcylation, and ribosylation. Additional, AMPK regulates DNA methylation mediated by influencing the SAM/SAH ratio, TET regulation and inhibition of DNMT1. Technological advances in data analysis continue to reveal the remarkable and diverse roles of AMPK in signaling pathways and epigenetic regulation, providing opportunities to exploit novel therapies relating to health and disease.

## Abbreviations

AMPK	AMP-activated protein kinase
CPT1C	carnitine palmitoyltransferase 1C
p21	cyclin-dependent kinase inhibitor
p53	TP53 or tumor protein
HAT	histone deacetylases
acetyl-CoA	acetyl coenzyme-A
HDAC	histone deacetylases
AICAR	5-Aminoimidazole-4-carboxamide ribonucleotide
ACC	acetyl-CoA carboxylase
ACSS2	acetyl-CoA synthetase short-chain family member 2
TFEB	transcription factor EB
SIRT	sirtuin
NAD+	nicotinamide adenine dinucleotide
βOHB	β-hydroxybutyrate
RBBP7	retinoblastoma binding protein 7
PGC-1α	peroxisome proliferator-activated receptor gamma coactivator-1α
Tfam	transcription factor A
NRF	nuclear respiratory factors
UCP	uncoupling proteins
SAM	S-adenosyl methionine
GLUT-4	glucose transporter type 4
KDM2A	lysine-specific demethylase 2A
KDM5	demethyltransferase lysine demethylase 5
LSD1	lysine-specific histone demethylase-1
PRC2	polycomb repressive complex 2
COMPASS	complex proteins associated with Set1
EZH2	enhancer of zeste homolog 2
Cdx2	caudal type homeobox2
SLC5A8	solute carrier family 5 member 8
*O*-GlcNAc	O-linked *N*-acetylglucosamine
OGT	*O*-GlcNAc transferase
UDP-GlcNAc	uridine diphosphate *N*-acetylglucosamine
HBP	hexosamine biosynthesis pathway
ART	mono-ADP ribosyltransferase
PTM	Post translational modification
PARP	poly-(ADP-ribose) polymerase
Bcl-6	B-cell Lymphoma protein 6
CpG	cytosine-guanine dinucleotides
DNMT	DNA methyltransferase
MBD	methyl-CpG binding domain proteins
5-mC	5-methycytosine
5-hmC	5-hydroxymethylcytosine
5-fC	5-formylcytosine
5-caC	5-carboxylcytosine
TETs	ten-eleven translocation hydroxylases
SAH	S-adenosylhomocysteine
SHMT2	serine hydroxymethyltransferase 2
SAHH	S-adenosylhomocysteine hydrolase
COX4I	cytochrome C oxidase subunit 4I1
FABP3	fatty acid binding protein 3
PPARδ	peroxisome proliferator-activated receptor δ
TCA	tricarboxylic acid
IDH2	isocitrase dehydrogenase 2
IP-Seq	immunoprecipitation sequencing
ATAC-seq	assay for transposase-accessible chromatin using sequencing
GEO	gene expression omnibus
SRA	sequence read archive
dbSNP	single nucleotide polymorphism database
3DGD	3D genome database
